# Accelerating Stroke MRI With Diffusion Probabilistic Models Through Large‐Scale Pre‐Training and Target‐Specific Fine‐Tuning

**DOI:** 10.1002/mrm.70501

**Published:** 2026-07-09

**Authors:** Yamin Arefeen, Sidharth Kumar, Steven Warach, Hamidreza Saber, Jonathan Isaac Tamir

**Affiliations:** ^1^ Chandra Family Department of Electrical and Computer Engineering The University of Texas at Austin Austin Texas USA; ^2^ Department of Imaging Physics MD Anderson Cancer Center Houston Texas USA; ^3^ Dell Medical School Department of Neurology The University of Texas at Austin Austin Texas USA; ^4^ Dell Medical School Department of Neurosurgery The University of Texas at Austin Austin Texas USA; ^5^ Dell Medical School Department of Diagnostic Medicine The University of Texas at Austin Austin Texas USA; ^6^ Oden Institute for Computational Engineering and Sciences The University of Texas at Austin Austin Texas USA

**Keywords:** accelerated MRI, clinical translation, diffusion probabilistic models, pre‐training and fine‐tuning, stroke MRI

## Abstract

**Purpose:**

To develop a data‐efficient strategy for accelerated MRI reconstruction with Diffusion Probabilistic Generative Models (DPMs) that enables faster scan times in clinical stroke MRI when only limited fully‐sampled data are available.

**Methods:**

Our simple training strategy first pre‐trains a DPM on a large, diverse collection of publicly available fastMRI brain data and then fine‐tunes on a small target dataset using carefully selected learning rates and fine‐tuning durations. The approach is evaluated on controlled fastMRI experiments and on clinical stroke MRI data with a blinded clinical reader study.

**Results:**

DPMs pre‐trained on 4000 non‐FLAIR subjects and fine‐tuned on FLAIR data from only 20 target subjects achieve reconstruction performance comparable to models trained with substantially more target‐domain FLAIR data across multiple acceleration factors. Moderate fine‐tuning with a reduced learning rate yields improved performance, while insufficient or excessive fine‐tuning degrades reconstruction quality. In a blinded reader study of 80 subjects at a single clinical site, images reconstructed from 2× accelerated data using the proposed approach are rated comparably to standard‐of‐care on the image quality and structural delineation metrics defined in this work.

**Conclusion:**

Large‐scale pre‐training combined with targeted fine‐tuning can enable DPM‐based MRI reconstruction for our data‐constrained, accelerated clinical stroke MRI application. In the single‐site settings evaluated here, the proposed approach reduces the need for large application‐specific datasets while maintaining clinically acceptable image quality, providing preliminary evidence for pre‐trained and fine‐tuned diffusion models as a strategy for accelerated MRI in targeted applications.

## 1 Introduction

Machine learning currently achieves state‐of‐the‐art performance in reconstructing MRI images from under‐sampled data for many anatomies, contrasts, and datasets [1, 2, 3, 4, 5]. However, these models are typically trained on datasets specific to the application of interest and are applied without modification for inference. While effective given sufficient data, this approach degrades in settings with limited data to train the models for the desired application [1]. In other domains, pre‐training on large, diverse datasets followed by adaptation to target applications has proven effective for overcoming data scarcity [6, 7]. This strategy has been successfully applied across natural language processing [8], vision [9], segmentation [10], fluorescence microscopy [11], and various healthcare applications [12, 13, 14].

As a motivating example, medical imaging is critical to diagnosis of ischemic stroke in patients who come to the emergency room presenting symptoms; many sites use computed tomography (CT) due to its effectiveness, widespread availability, and relatively short scan times [15]. However, MRI offers improved sensitivity when detecting ischemic stroke and more precise localization of the ischemic lesion [16]. Despite these advantages, MRI typically requires longer scan times and is more susceptible to degradation from patient motion, which can delay image acquisition and, consequently, time to treatment. Machine learning‐based acceleration methods could mitigate these limitations by reducing scan duration and motion sensitivity [17], but limited stroke specific training data precludes their application.

Several techniques have been proposed to train machine learning reconstruction models in settings with limited, fully‐sampled data. Some methods train reconstruction models on just the under‐sampled scan itself by leveraging calibration regions [18, 19], implicit regularization imposed by neural network structure [20, 21], and self‐supervised learning through k‐space splitting [22]. However, these methods fall short of state‐of‐the‐art reconstruction quality offered by methods trained on large amounts of high quality data [1, 3, 4]. Other self‐supervised training strategies instead utilize under‐sampled or corrupt images for training [23, 24, 25, 26, 27, 28, 29] but still require a large corpus of data. In addition, these methods implement end‐to‐end reconstructions mapping under‐sampled data to images given a specific acquisition model. Diffusion‐based reconstruction methods have also been developed for limited or mismatched target‐domain data. Several methods adapt the diffusion prior per‐scan at inference using only the measurement, via full‐weight updates [30], low‐rank residual pathways [31], or a deep‐image‐prior formulation [32], while others train self‐supervised unrolled diffusion models directly on under‐sampled k‐space [33]. Our approach targets a different data‐availability regime: we adapt an unconditional DPM once via offline fine‐tuning on a small fully‐sampled target cohort, then apply standard posterior sampling at inference.

Motivated by data limitations in our stroke application, we propose a method to train diffusion probabilistic generative models (DPMs) for accelerated MRI reconstruction given a small dataset for the contrast of interest. Inspired by similar approaches for large language models [34], our simple but effective strategy first trains a DPM on a large, public dataset of diverse MR images and then fine‐tunes the DPM on the smaller, fully‐sampled dataset from the target application, with the new contrast assigned to a one‐hot index in the conditioning embedding reserved at pre‐training. During fine‐tuning, we select hyperparameters to adapt the model effectively without overfitting and retaining the benefits of pre‐training on a large, diverse dataset [35]. We select DPMs for the reconstruction model because they are agnostic to the acquisition forward model [36, 37, 38, 39, 40, 41, 42, 43, 44, 45]. Thus unlike end‐to‐end methods which require the larger external dataset and the smaller target dataset to be acquired with the same measurement model (sampling pattern, coil geometry, etc.) [1], a DPM pre‐trained on the larger dataset can be adapted to smaller target datasets with different acquisition models. While the individual components of our approach, including score‐based diffusion models, posterior sampling, and the pre‐train then fine‐tune paradigm, have been proposed and validated in other settings, the contribution of this work lies in their careful integration and systematic evaluation for accelerated stroke MRI reconstruction where fully‐sampled training data are scarce.

First, we evaluate the proposed approach on fastMRI [3] by initially pre‐training a DPM on $T_{1}$, $T_{2}$, and $T_{1}$‐post brain scans from 4000 subjects. To simulate access to limited data on a target application, we then finetune the DPM on FLAIR data from just 20 subjects of fastMRI. Across a range of acceleration factors, DPMs trained with our method achieve comparable performance to DPMs trained with 344 target FLAIR subjects. Our experiments also characterize the sensitivity of reconstruction quality to inference hyperparameters for DPM‐based MRI reconstruction and showcase application on prospectively under‐sampled data.

Next, we apply our method to the motivating setting of stroke MRI. We initially pre‐train a DPM on $T_{1}$, $T_{2}$, $T_{1}$‐post, and FLAIR brain scans from approximately 4000 fastMRI subjects and then fine‐tune the DPM on data from 25 stroke patients from our local hospital. On 5 held‐out test patients, the proposed fine‐tuning strategy improves reconstruction of SWI, MPRAGE, DWI, and FLAIR images from 2× further under‐sampled stroke data.

Finally, we conduct a clinical validation study in which two radiologists experienced in stroke MRI compared SWI, MPRAGE, DWI, and FLAIR images reconstructed from retrospective, 2× under‐sampled data using our method to standard‐of‐care images. The reader study indicates that, for the image quality and structural delineation metrics defined in this work, images reconstructed by the DPM pre‐trained on fastMRI and fine‐tuned on local stroke data are rated comparably to the standard‐of‐care in our single‐site cohort.

## 2 Methods

### 2.1 Diffusion Probabilistic Generative Models for MRI Reconstruction

We model the multi‐coil MRI acquisition as y=PFSx+n where y are the acquired measurements, P is the under‐sampling operator, F is the Fourier Transform, S is the multi‐coil operator, n is additive gaussian noise, and x is the image to reconstruct. DPMs reconstruct accelerated MRI acquisitions by first learning the underlying probability distribution of the target MR contrast and anatomy, and then guiding the reconstruction toward solutions that both match the data and are statistically likely. Specifically, we use score‐based diffusion models where a neural network $G_{\theta }$ approximates the score function $\nabla_{x_{t}}\log p_{t}\left(x\right)$, where $p_{t}\left(x\right)$ denotes image distribution perturbed by gaussian noise with standard deviation $\sigma_{t}$ [36, 41, 42, 46]. Equipped with the score function approximation, images can be reconstructed from under‐sampled data by approximately sampling from the posterior p(x|y) with posterior sampling algorithms [43].

In this work, we combine the prior sampling formulation from Reference [46] with Diffusion Posterior Sampling [38] to approximately sample from p(x|y) in accelerated MRI. Specifically, we solve the following ordinary differential equation (ODE):

(1) $$dx=\left[\frac{\dot{s}\left(t\right)}{s\left(t\right)}x-s{\left(t\right)}^{2}\dot{\sigma }\left(t\right)\sigma \left(t\right)\nabla_{x}\log p\left(\frac{x}{s\left(t\right)}|y;\sigma \left(t\right)\right)\right]\mathrm{dt}.$$

Setting s(t)=1 and σ(t)=t based on Reference [46], applying Baye's rule to separate the posterior score into the sum of the log‐likelihood and prior score, and inserting the DPM that approximates the prior score function and the closed‐form expression for the likelihood score [38, 39] results in the following ODE,

(2) $$dx=\left[-t\left(\nabla_{x}{\left\|\mathrm{PFS}\tilde{x}\left(x\right)-y\right\|}_{2}^{2}+D_{\theta }\left(x,t\right)\right)\right]\mathrm{dt}.$$

We apply the approximation $\tilde{x}\left(x\right)=E\left[x_{0}|x\right]$ since the log‐likelihood score is only known at t=0 [38].

### 2.2 DPM Details

All DPMs in this work use the U‐Net style architecture from Reference [36], as implemented by Reference [46], and we adopt a modified version of the architecture that supports inputs with varying matrix sizes [47]. Initial training on the external dataset and fine‐tuning on the target dataset applied the “EDM” training loss, data augmentation, optimizer choice, and noise level scheduling. More specifically, the EDM training loss is a weighted denoising score‐matching objective, which we adopted exactly as presented in Reference [46], including its loss weighting and noise‐level distribution, without modification across image contrasts. We similarly used the EDM‐recommended exponential moving average half‐life of 0.1, no dropout, and preconditioning configuration from table 1 of Reference [46]. Data augmentation followed EDM [46] without modification, applying horizontal and vertical flips together with geometric transformations including scaling, rotation, and translation. Each augmentation was applied independently with probability 0.12, and the same augmentation settings were used across all image contrasts. Training used the Adam optimizer with $\beta_{1}=0.9$, $\beta_{2}=0.999$, and $\epsilon =10^{-8}$. Pre‐training used a learning rate of $10^{-4}$, and contrast‐specific fine‐tuning learning rates are reported in the corresponding experimental sections. The noise‐level schedule followed the DDPM++ parameterization in table 1 of Reference [46]. The U‐Net has approximately 31 million trainable parameters.

To accommodate datasets with heterogeneous contrasts, we simultaneously train on all available data by conditioning the model on contrast type [47]. Each image contrast type in the training set is assigned a one‐hot vector, which is passed through a small fully‐connected neural network to produce an embedding. The embedding vector has dimensionality 512 and is supplied to each U‐Net block as an additional conditioning input, rather than concatenated as an extra channel to the noisy image input, allowing the model to adapt its behavior based on the contrast (see Supporting Information and Figure S4 for additional implementation details). The one‐hot vector itself has dimension 9, intentionally larger than the number of contrasts present at pre‐training. Indices not associated with a pre‐training contrast remain inactive throughout pre‐training and are reserved for new target contrasts introduced at fine‐tuning. The DPM and embedding network are trained jointly.

### 2.3 Posterior Sampling Details

Algorithm 1 outlines the posterior sampling procedure utilized in this work for accelerated MRI reconstruction adapted from the ODE prior sampling procedure used in Reference [46] and the log‐likelihood approximation proposed by Reference [38]. The noise levels, ${\hat{\sigma }}_{i}$ follow the recommended schedule from Reference [46], and N denotes the number of sampling steps. Since this posterior sampling is approximate, $\zeta_{i}$ is introduced as a tunable hyperparameter that balances data consistency and learned prior score at each iteration. On an NVIDIA A100 GPU, the current implementation requires approximately 45 s to reconstruct a single slice.

ALGORITHM 1 **Diffusion posterior sampling (DPS).**

**Require:** $G_{\theta }\left(x;\sigma \right),N,\left\{\zeta_{i=0}^{N-1}\right\},{\left\{{\hat{\sigma }}_{i}\right\}}_{i=0}^{N-1}$
 **sample** $x_{t_{0}}∼\mathbb{N}\left(0,I\right)$
 **for** i∈{0,…,N−1} **do**
   $\hat{s}=\frac{G_{\theta }\left(x;\sigma \right)-x_{i}}{\sigma }$
   ${\hat{x}}_{0}=\frac{1}{\sqrt{\alpha_{i}}}\left(x_{i}+\left(1-{\bar{\alpha }}_{i}\hat{s}\right)\right)$
   n∼ℕ(0,I)
   ${\tilde{x}}_{p}=\frac{\sqrt{\alpha_{i}}\left(1-{\bar{\alpha }}_{i-1}\right)}{1-{\bar{\alpha }}_{i}}x_{i}+\frac{\sqrt{{\bar{\alpha }}_{i-1}}\beta_{i}}{1-{\bar{\alpha }}_{i-1}}{\hat{x}}_{0}$
   ${\tilde{x}}_{\mathrm{DC}}=\nabla_{x_{i}}{\left\|y-A\left({\hat{x}}_{0}\right)\right\|}_{2}^{2}$
   $x_{i+1}={\tilde{x}}_{p}-\zeta_{i}{\tilde{x}}_{\mathrm{DC}}+\sigma_{i}n$
**end for**

### 2.4 Proposed Training Strategy

**FIGURE 1:**
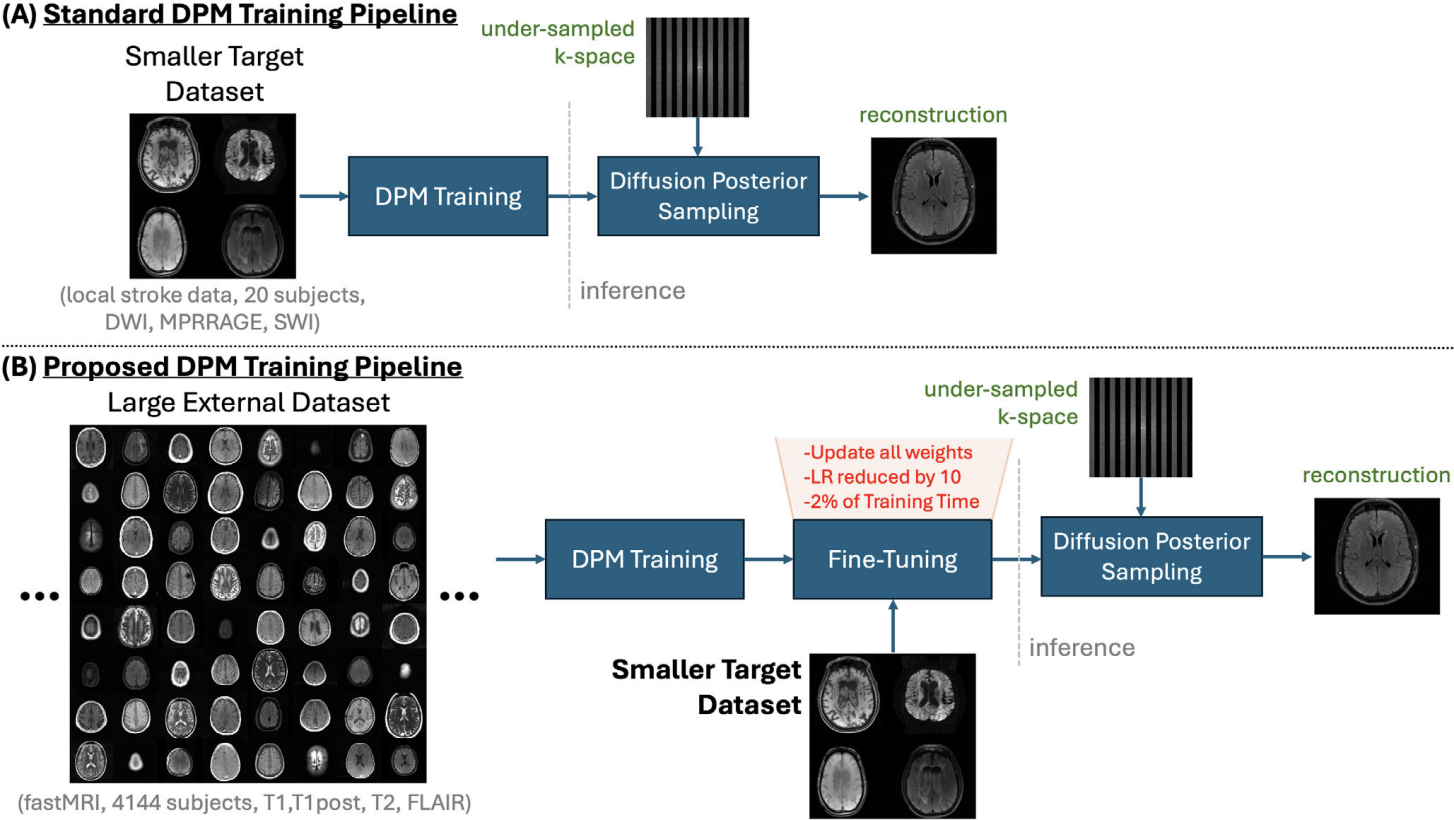
(A) The standard DPM training pipeline trains exclusively on the target‐domain dataset, but if the target dataset is small, this can lead to degraded performance. (B) The proposed training pipeline mitigates data scarcity by first pre‐training the DPM on a large, diverse external MRI dataset, followed by fine‐tuning on a small target‐domain dataset using a reduced learning rate and limited number of epochs. The fine‐tuned model is then used for posterior sampling‐based reconstruction from under‐sampled k‐space.

Inspired by strategies for foundation and large language models [34, 48] and related pre‐training and transfer‐learning approaches for MRI reconstruction [49, 50], we propose a simple and effective training approach to train DPMs for accelerated MRI when only limited target‐domain data is available. Our strategy involves two stages: (i) pre‐training on a large, diverse external dataset, like fastMRI [3], covering multiple MRI contrasts; and (ii) fine‐tuning on the smaller, target dataset, updating all model weights. To mitigate overfitting during fine‐tuning, we reduce the learning rate by an order of magnitude and train for a small number of epochs, corresponding to roughly 2% of pre‐training time. In addition, we assign the target contrast to one of the reserved one‐hot indices from Section 2.2 that was inactive during pre‐training, activating that dimension for the first time at the start of fine‐tuning, and update the weights of the embedding network during fine‐tuning. This reserved‐index design lets a new target contrast be introduced at fine‐tuning without modifying the network architecture. Figure 1 compares (A) standard and (B) the proposed training strategies for DPMs.

### 2.5 Controlled Experiments With FastMRI

To validate the proposed training strategy, we use the multi‐coil brain dataset from FastMRI [3]. Our dataset included Axial $T_{2}$ from 2678 subjects, Axial $T_{1}$ from 498 subjects, postcontrast Axial $T_{1}$ from 949 subjects, and Axial FLAIR from 344 subjects (of 411 total Axial FLAIR scans in fastMRI). These fastMRI Axial FLAIR data span a heterogeneous range of acquisition settings across multiple Siemens platforms (Aera, TrioTim, Skyra, Prisma, and Biograph mMR) at both 1.5 and 3 T, with TR 9000 ms, TE 76–126 ms, TI 2500 ms, refocusing flip angle 143°–150°, a turbo spin‐echo readout, reconstructed matrix 256–512, 200–230 mm field of view (in‐plane resolution on the order of 0.5 mm), and 3–5 mm slice thickness. We focused on reconstructing Axial FLAIR images, simulating a low‐data regime by limiting the FLAIR training set to only 20 subjects sampled from these 344. We deliberately exclude FLAIR from pre‐training in this controlled experiment to create a clean separation between the non‐FLAIR pre‐training source and the FLAIR target. This design lets a model trained on all 344 FLAIR subjects serve as an upper‐bound reference (Methods 1–3) against which fine‐tuning on only 20 FLAIR subjects (Methods 4–6) can be directly compared, and lets us study fine‐tuning hyperparameters against a verifiable reference. In the clinical stroke application (Section 2.7), where target data are genuinely scarce, all available fastMRI data including FLAIR are instead used for pre‐training. All k‐space data were pre‐whitened and re‐sized to 320×320 matrix size, and training was performed on fully‐sampled, coil‐combined images normalized to the 99th percentile of the maximum absolute value. For testing, 500 random Axial FLAIR slices were selected from the remaining 67 held‐out fastMRI subjects not used during training.

We first investigated the effect of learning rate and training time during fine‐tuning. A DPM was pre‐trained on the larger external dataset with a learning rate of $10^{-4}$ for 625,000 epochs, and then fine‐tuned on the 20‐subject FLAIR dataset with learning rates of $\left\{5\times 10^{-4},1\times 10^{-4},5\times 10^{-5},5\times 10^{-5}\right\}$ and epochs of {0,625,1250,1875,2500}.

In addition, we compared our proposed training paradigm to alternative methods.

- *Method 1*: Trained a DPM on the full dataset, using all 2678 Axial $T_{2}$, 498 Axial $T_{1}$, 949 Postcontrast Axial $T_{1}$, and 344 Axial FLAIR subjects.
- *Method 2*: Pre‐trained on $T_{1}$, $T_{2}$ and Postcontrast $T_{1}$ data and fine‐tuned on all 344 FLAIR subjects.
- *Method 3*: Trained only on the 344‐subject FLAIR dataset.
- *Method 4 (Proposed)*: Pre‐trained on all $T_{2}$, $T_{1}$, and $T_{1}$ postcontrast data and then fine‐tuned on 20 FLAIR subjects with reduced learning rate and shorter training time.
- *Method 5*: Trained solely on the 20‐subject FLAIR dataset.
- *Method 6*: Trained jointly on the full external dataset and the 20‐subject FLAIR dataset without fine‐tuning.

Methods 1–3 serve as performance upper bounds, as they use extensive target contrast (FLAIR) data. Methods 4–6 represent the constrained data regime, relying on large external datasets and limited target‐domain data.

All methods were evaluated on the 500 validation FLAIR slices using under‐sampling rates of R={3,4,5,6,7,8} with no auto‐calibration (ACS) data. Retrospective under‐sampling used equispaced Cartesian masks.

### 2.6 Evaluating Posterior Sampling Hyperparameters

We evaluated the effect of hyperparameter selection in Algorithm 1 for approximate posterior sampling. In particular, the data consistency weighting ζ is often set to be inversely proportional to the norm of the data consistency error [38], while the number of steps N trades off between quality and computation. Given a DPM trained for FLAIR reconstruction with the proposed method, we compared reconstruction performance for different choices of ζ between 1/2 and 5 and N between 200 and 300 when reconstructing data at R={3,4,5,6,7,8}.

### 2.7 Application to Clinical Stroke MRI

In collaboration with our local hospital's medical school, under IRB approval and informed consent, we collected a small dataset of clinical stroke MRI from 30 patients on a 3 T Siemens Vida scanner. Each subject underwent a standard‐of‐care stroke imaging protocol following triage, including T2‐FLAIR, MPRAGE, SWI, and DWI. The standard‐of‐care data were reconstructed with SENSE‐based parallel imaging using coil maps calibrated with BART [51]. The list below summarizes the acquisition parameters:

- T2‐FLAIR: 0.5×0.5mm in‐plane resolution, 5mm slice thickness, 9000ms TR, 82–84ms TE, 2500ms TI, $150^{{}^{\circ}}$ refocusing flip angle, turbo spin‐echo readout.
- MPRAGE: 1.5×1.5×1.5mm resolution, 240×240×168mm FOV, 2500ms TR, 4.17ms TE, 1210ms TI, $8^{{}^{\circ}}$ flip angle.
- SWI: 1.0×1.0mm resolution, 250×211mm FOV, 2.5mm slice thickness, 27ms TR, 20ms TE, $15^{{}^{\circ}}$ flip angle, 3D gradient‐echo readout.
- DWI: 0.8×0.8mm resolution, 250×250mm FOV, 30 slices, 5.0mm slice thickness, *b*‐values: $0,1200s/{\mathrm{mm}}^{2}$, 6100ms TR, 111ms TE, $90^{{}^{\circ}}$ flip angle, echo‐planar readout.

The fastMRI FLAIR data used for pre‐training span a heterogeneous range of acquisition settings, reported in Section 2.5. The clinical T2‐FLAIR protocol above (3 T Siemens Vida, turbo spin‐echo, 0.5×0.5 mm in‐plane, 5 mm slice) falls largely within this range: the 30‐subject clinical cohort used for fine‐tuning and testing matches the fastMRI FLAIR timing closely (TR 9000 ms, TI 2500 ms), while the 80‐subject reader‐study cohort additionally spans TR 5000–9000 ms and TI 1800–2500 ms. The principal distributional differences are therefore that the clinical target is acquired exclusively at 3 T on the Vida platform, which is not represented among the fastMRI FLAIR scanners, and that the reader‐study cohort exhibits a range of repetition and inversion times beyond the fixed TR and TI of the fastMRI FLAIR data.

To evaluate our method in this clinical setting, we first pre‐trained a DPM on all Axial FLAIR, Axial $T_{2}$, Axial $T_{1}$, and Axial $T_{1}$ post contrast data available from the 4125 subjects in fastMRI. Then, we fine‐tuned the DPM seperately for each clinical sequence (T2‐FLAIR, MPRAGE, SWI, DWI) using 20 stroke patients from our local dataset. Fine‐tuning was performed with a reduced learning rate and limited training duration as described in Section 2.4.

An additional 5 stroke subjects were used for validation to select hyper‐parameters for fine‐tuning and posterior sampling. More specifically, the 30 stroke subjects are partitioned into 20 fine‐tuning, 5 validation, and 5 test subjects, and this partition is fixed before any model is trained. For each clinical sequence (T2‐FLAIR, MPRAGE, SWI, DWI), we sweep fine‐tuning learning rates and numbers of epochs as well as the posterior‐sampling weight ζ and number of steps N, evaluating each configuration on the 5‐subject validation cohort. The configuration that minimizes the mean NRMSE on the validation cohort is selected per sequence (Figures 6 and 7 and Figure S1) and frozen before any test reconstruction is performed. Combined with the reduced learning rate and limited fine‐tuning duration of Section 2.4, this protocol is intended to mitigate overfitting to the 20‐subject fine‐tuning cohort. Fine‐tuned DPMs then reconstructed retrospectively under‐sampled data (accelerations around R≈4, i.e., 2× further under‐sampling beyond the standard‐of‐care acceleration of R≈2) on the remaining 5 test subjects.

### 2.8 Clinical Stroke Reader Study

We acquired additional clinical stroke MRI data from 80 subjects at our local hospital with IRB approval and informed consent to conduct a blinded reader study. Each subject was scanned using the standard‐of‐care stroke protocol, including T2‐FLAIR, MPRAGE, SWI, and DWI acquisitions obtained at an acceleration factor of approximately R≈2 and reconstructed using SENSE‐based parallel imaging. To evaluate accelerated imaging, the same data were retrospectively under‐sampled by an additional factor of two with an equispaced (uniform) Cartesian pattern, resulting in a total effective acceleration of approximately R≈4, and reconstructed using diffusion probabilistic models (DPMs) fine‐tuned with the proposed training strategy.

For each of the 80 subjects, the reconstructed images corresponding to the standard‐of‐care and proposed accelerated protocols were grouped by subject, blinded, and randomized, yielding a total of 160 image sets for evaluation. Importantly, each image set comprised contrasts from the stroke protocol, and readers assessed the image set as a whole rather than individual sequences in isolation. Reader H.S. evaluated all 160 image sets, while reader S.W. evaluated 42 image sets (corresponding to 21 subjects) due to time constraints; H.S. and S.W. have 11 and 30 years of clinical experience, respectively.

Each image set was scored for structural delineation of white matter, gray matter, and ventricles, as well as for image quality metrics including signal‐to‐noise ratio (SNR), contrast, sharpness, artifacts, and overall image quality. All evaluations were performed using a five‐point ordinal scale where [1←repeat scan needed,2←limited,3←diagnostic,4←good,5←outstanding]. Statistically significant differences in metrics was assessed using paired two‐sided Wilcoxon signed‐rank tests and inter‐rater reliability was measured with the Cohen's kappa coefficient.

### 2.9 Experiments on Prospectively Under‐Sampled Acquisitions

Noting that our clinical evaluation involved retrospective subsampling, we further evaluated our method with prospective under‐sampling. We acquired SWI and FLAIR data from a healthy subject under IRB approval and informed consent using the parameters of the typical stroke protocol at our local hospital. Two acquisitions were performed: one at the standard‐of‐care acceleration rate, R=1.7, and another at a prospective acceleration rate of R=3.75. We applied the proposed method, pre‐trained on all $T_{2}$, $T_{1}$, and postcontrast $T_{1}$ from fastMRI and fine‐tuned on our FLAIR and SWI stroke data, to reconstruct the data prospectively under‐sampled to R=3.41 and R=3.75, respectively and to reconstruct the R=1.75 under‐sampled data after it was further retrospectively under‐sampled by R=3.52 and R=3.75, respectively. Reconstructions on both datasets were compared to a L1‐wavelet baseline implemented with BART [51].

## 3 Results

### 3.1 Controlled Experiments With FastMRI

**FIGURE 2:**
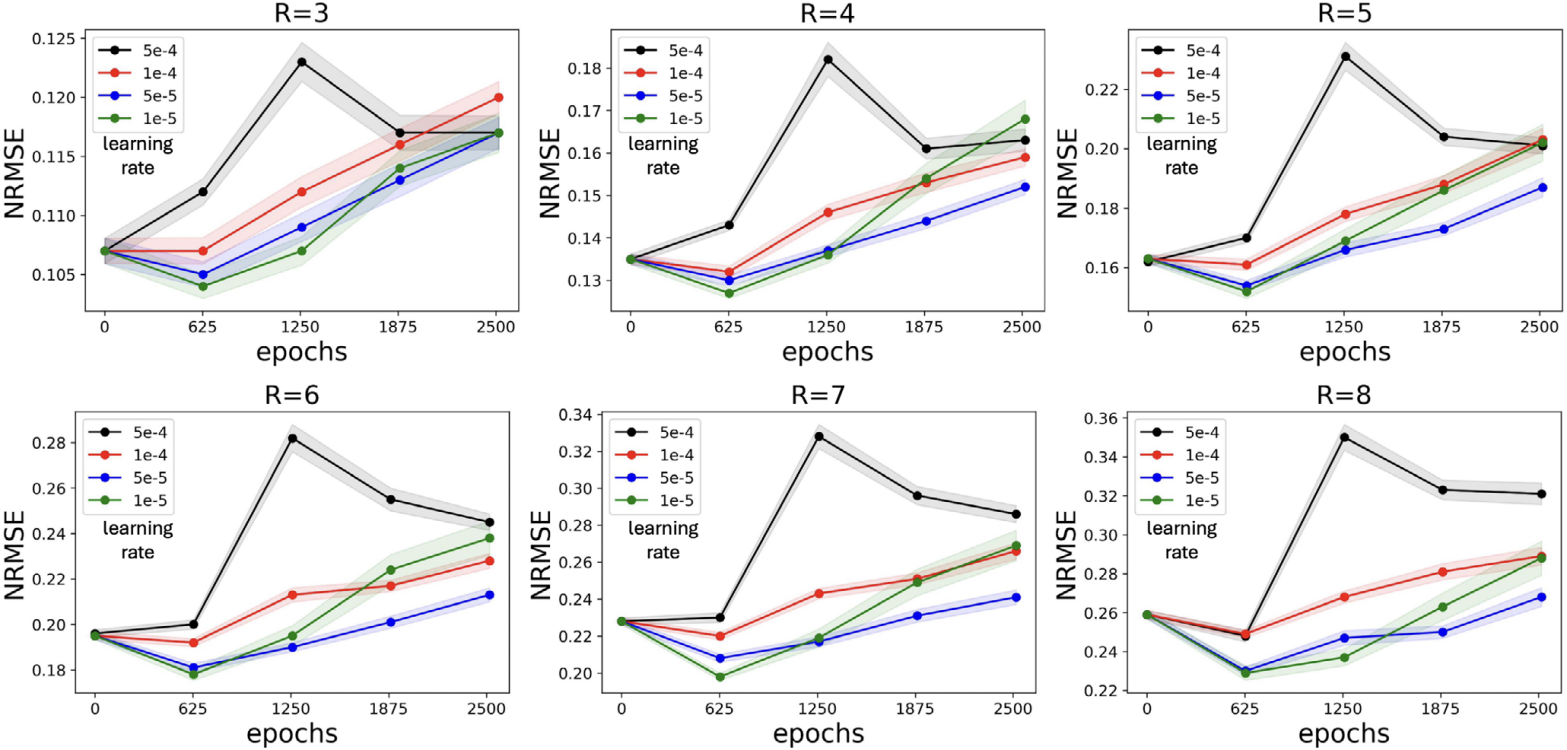
Quantitative results from the controlled fastMRI experiments where DPMs were fine‐tuned on 20 subjects to reconstruct fastMRI flair data. NRMSE on 500 validation slices as a function of fine‐tuning duration (epochs) is plotted for different learning rates and acceleration factors. Across all acceleration factors, moderate fine‐tuning improves reconstruction quality relative to no fine‐tuning, while excessive fine‐tuning leads to degraded performance, particularly at higher learning rates.

Figure 2 quantitatively evaluates the proposed fine‐tuning method across different learning rates and training epochs, measured with normalized root mean squared error (NRMSE) at various acceleration factors on the 500 fastMRI FLAIR validation slices. Across all acceleration rates and learning rates, fine‐tuning for 650 epochs yields the lowest NRMSE, outperforming both no fine‐tuning (0 epochs) and longer (1250, 1875, 2500 epochs) fine‐tuning durations. Similarly, a learning rate of $1\times 10^{-5}$ achieves the best performance in comparison to higher learning rates.

**FIGURE 3:**
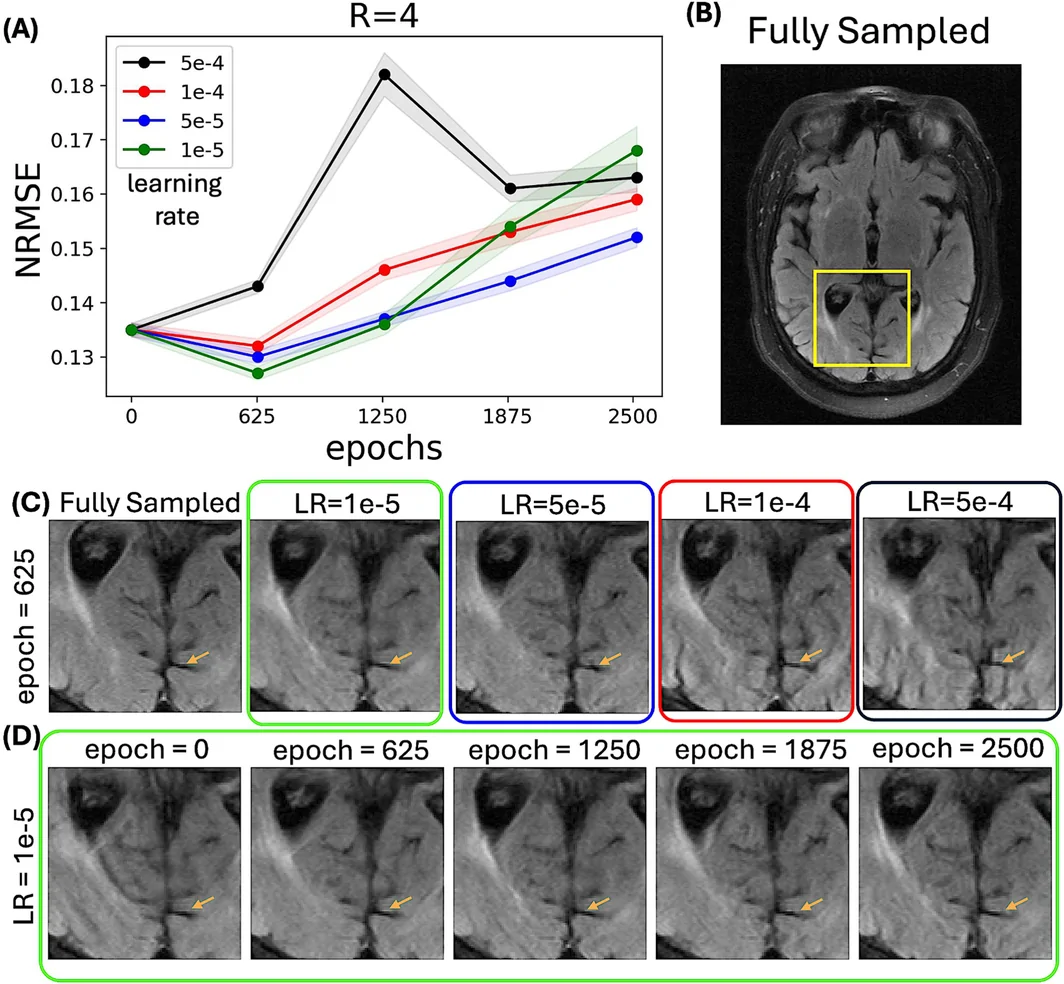
A representative example from the fastMRI FLAIR experiment. (A) Quantitative NRMSE as a function of fine‐tuning duration for different learning rates, highlighting that a comparatively smaller learning rate and fewer fine‐tuning epochs yields the best performance. (B) Fully sampled reference FLAIR image. (C) Zoomed reconstructions obtained using different learning rates with 650 fine‐tuning epochs, compared against the fully sampled reference. (D) Reconstructions obtained using a fixed learning rate of $1\times 10^{-5}$ across different fine‐tuning durations. Appropriate fine‐tuning hyperparameters lead to visibly improved structural fidelity and reduced artifacts, as highlighted by the orange arrows.

Figure 3 illustrates a representative example from the quantitative FLAIR experiment at R=4. As shown in (A), combining a learning rate of $1\times 10^{-5}$ and 650 epochs gives the best quantitative performance. (B) shows the fully‐sampled reference and (C) zooms into the reference and reconstructions under various learning rates and epochs. Qualitative improvements are observed, highlighted by the orange arrow, when the appropriate fine‐tuning hyperparameters are used.

**FIGURE 4:**
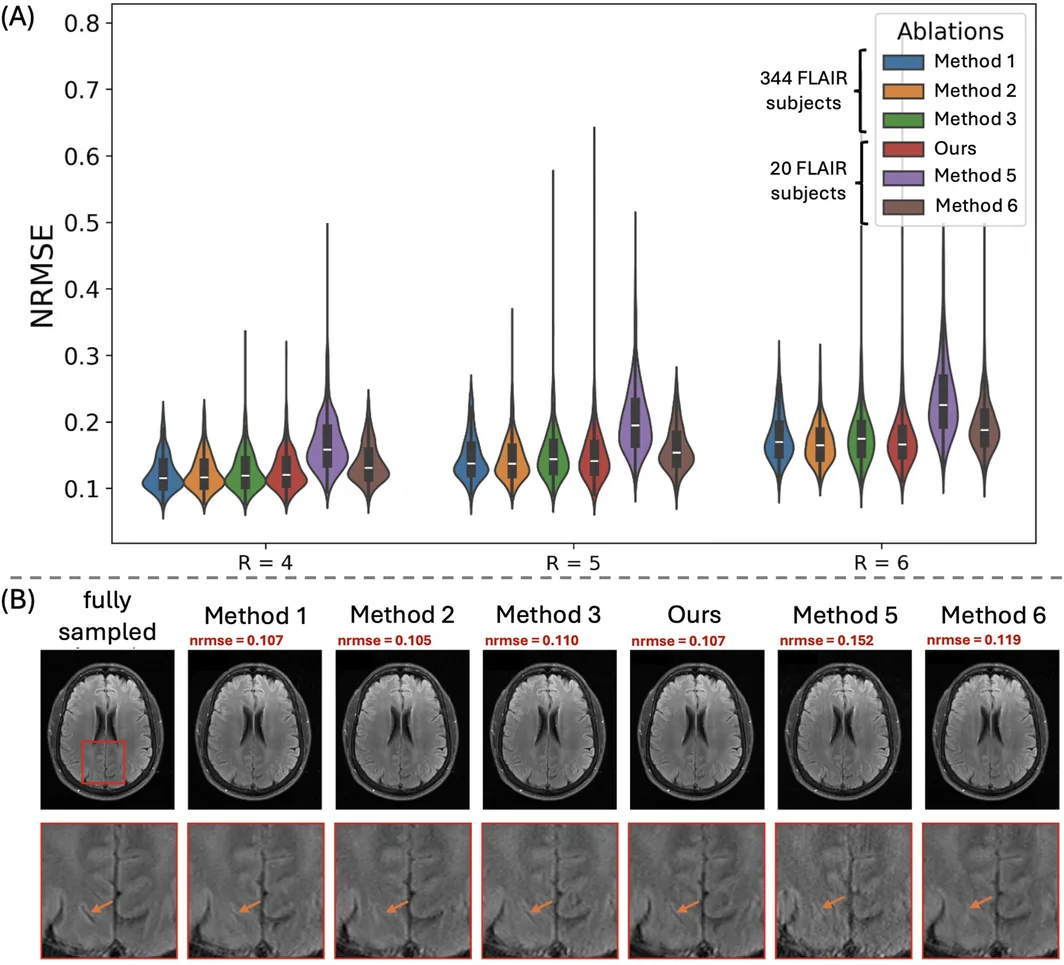
(A) DPMs are compared across methods with access to different amounts of target‐domain FLAIR data. The violin plots in (A) show the NRMSE distribution over the 500 test slices from the 67 held‐out fastMRI subjects (Section 2.5). Methods 1–3 are trained using FLAIR data from all 344 subjects, while Methods 5 and 6 are trained using 20 FLAIR subjects and the external dataset. The proposed approach (Method 4), pre‐trained on a large external dataset and fine‐tuned on 20 FLAIR subjects, achieves comparable NRMSE to Methods 1–3, despite using substantially less target domain data and consistently outperforms Methods 5, 6 trained on the same limited data. (B) Displays example reconstructions from this experiment. The NRMSE values reported in (B) are computed over the full displayed slice; for this representative example, the NRMSE differences among Methods 1–3 and the proposed approach do not fully reflect the localized visual differences highlighted by the orange arrows.

Figure 4 compares DPMs fine‐tuned using our method (650 epochs and $1\times 10^{-5}$ learning rate) on FLAIR data from 20 subjects to methods [1, 2, 3] which have access to FLAIR data from 344 subjects and methods (5, 6) with access to the same 20 FLAIR subjects and large external dataset. (A) plots quantitative reconstruction performance for acceleration rates R={4,5,6}. Our method achieves comparable performance to methods 1–3 which have access to much more FLAIR data and improved performance in comparison to methods 5, 6 with access to similar amounts of data. (B) shows example reconstructions from R=4 under‐sampled data. Quantitatively and qualitatively, as highlighted by the orange arrow, our method achieves comparable quality to methods 1–3 and outperforms methods 5 and 6.

**FIGURE 5:**
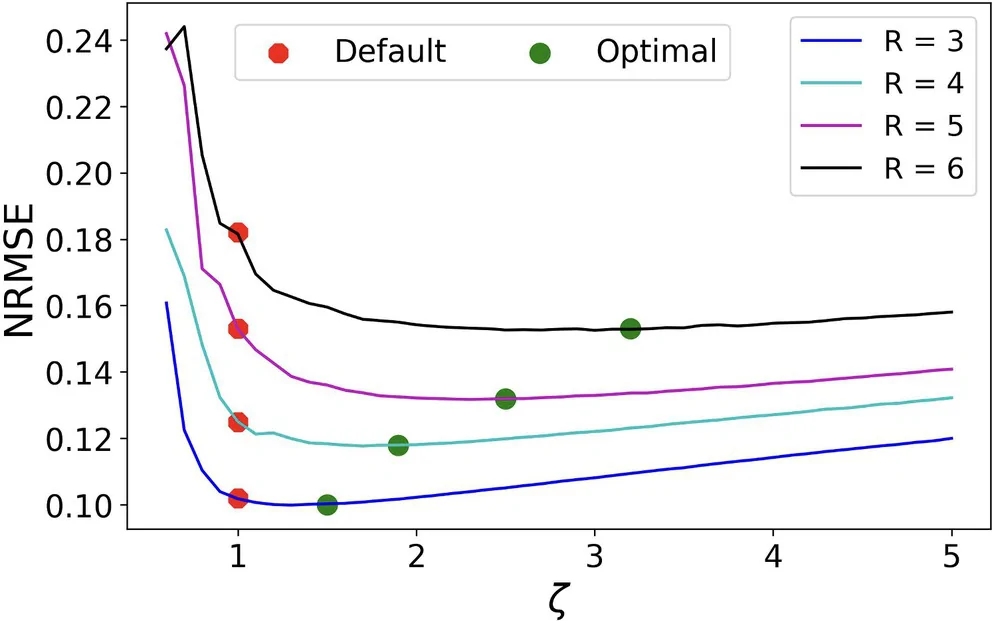
Using the best fine‐tuned DPM, average NRMSE on fastMRI FLAIR validation data is plotted as a function of the data‐consistency step size ζ for multiple acceleration factors. Red markers denote the default choice ζ=1, while green markers indicate the value of ζ that minimizes reconstruction error at each acceleration. The optimal ζ increases with acceleration.

Using our best fine‐tuned DPM, Figure 5 plots the average NRMSE across the validation samples for varying acceleration rates as a function of the data consistency step‐size ζ in Algorithm 1. Green dots highlight the typical default setting of ζ=1 and the red dots mark the value of ζ that achieves the lowest reconstruction error at each acceleration rate. The optimal choice of ζ increases with higher acceleration rate. Table S1 summarizes the best performing of ζ and number of posterior sampling steps N for each acceleration rate.

### 3.2 Application to Clinical Stroke MRI

**FIGURE 6:**
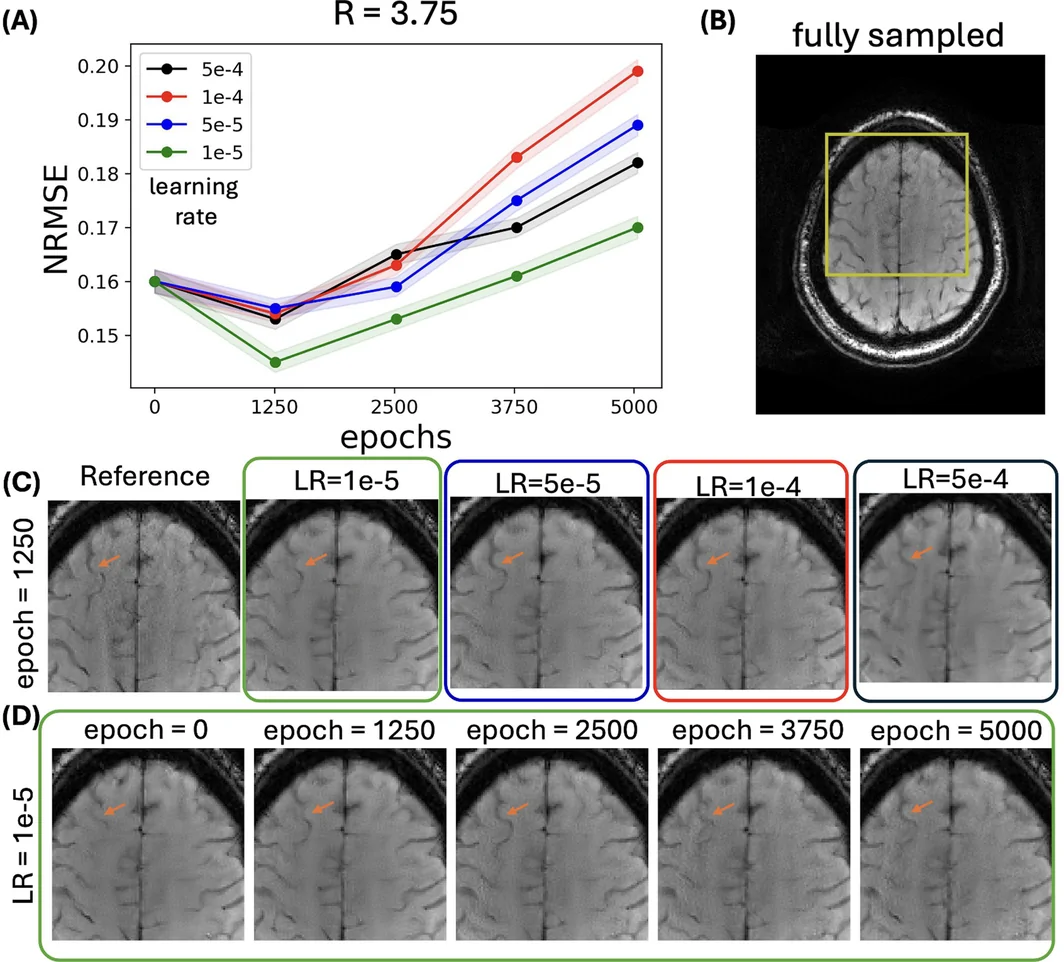
(A) NRMSE of SWI reconstructions averaged over five validation subjects from data retrospectively accelerated by R≈3.75. (B) Fully‐sampled reference. (C) Zoomed‐in reconstructions obtained with fixed epochs and varying learning rate and (D) fixed learning rate and varying epochs. Similar to the fastMRI FLAIR experiments, insufficient or excessive fine‐tuning duration and higher learning rates consistently result in worse reconstructions, demonstrating that the proposed training strategy is effective for clinical stroke SWI data.

Figure 6 evaluates DPMs fine‐tuned using the proposed technique with limited stroke data to reconstruct R≈3.75 SWI data from clinical stroke acquisitions. Similar to FLAIR in the fastMRI setting, the NRMSE plots in (A) averaged over the 5 validation subjects suggest that fine‐tuning for too few or too many epochs results in worse quantitative performance. In addition, higher learning rates yield worse performance for all training epochs. These results suggest that 1250 epochs and a learning rate of $1\times 10^{-5}$ yields improved performance for fine‐tuning a DPM to stroke SWI. (B) shows an example standard‐of‐care SWI image and (C) compares zoomed in views of this reference with reconstruction using DPMs fine‐tuned with different hyperparameters. Analysis on the 5 subject validation dataset found ζ=2.1 as the optimal data consistency weighting and was used for all test reconstructions.

**FIGURE 7:**
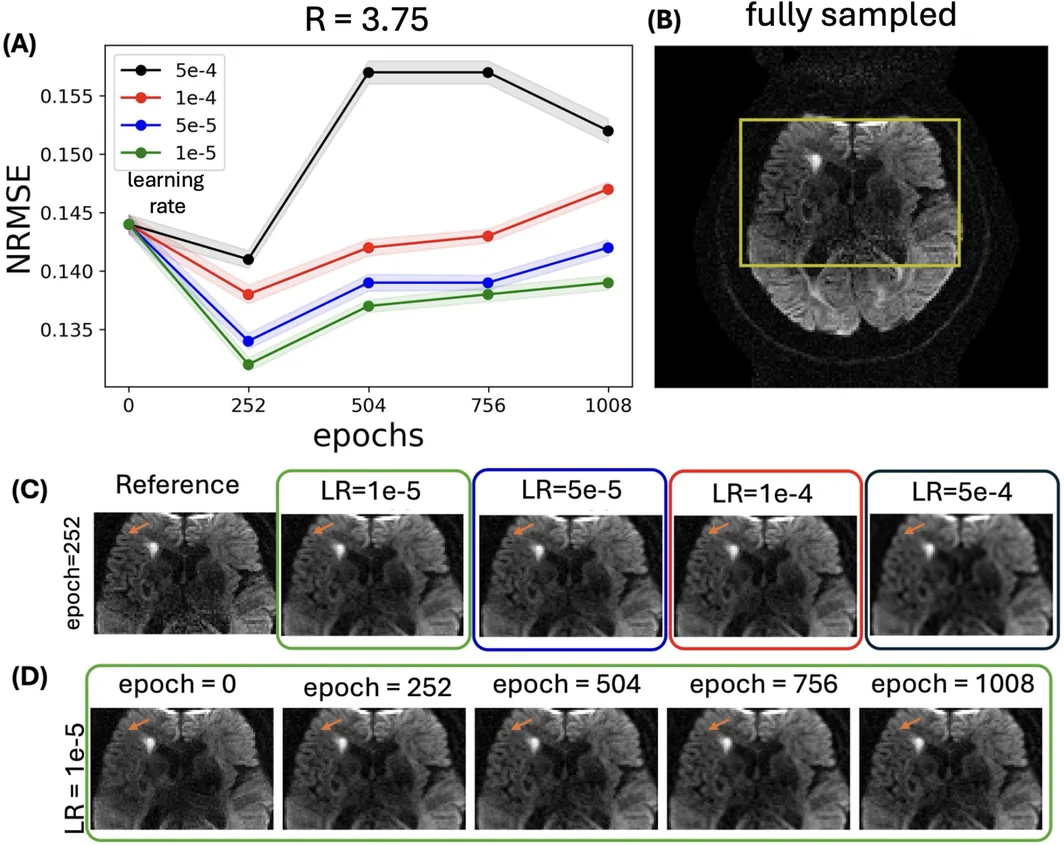
(A) NRMSE of DWI reconstructions averaged over five validation subjects from data retrospectively accelerated by R≈3.75. (B) Fully‐sampled reference. (C) Zoomed‐in reconstructions obtained with fixed epochs and varying learning rate and (D) fixed learning rate and varying epochs. Similar to the fastMRI FLAIR experiments, insufficient or excessive fine‐tuning duration and higher learning rates consistently result in worse reconstructions, demonstrating that the proposed training strategy is effective for clinical stroke DWI data.

Figure 7 presents fine‐tuning a DPM to reconstruct DWI acquired with *b*‐value $1200s/{\mathrm{mm}}^{2}$. (A) Fine‐tuning with 252 epochs and a learning rate of $1\times 10^{-5}$ yields the best performance. (B) shows an example standard‐of‐care DWI image and (C) compares zoomed in views of the reference and reconstructions using our fine‐tuned DPM with various hyperparameters.

Figure S1 analyzes the influence of fine‐tuning epochs and learning rate for the additional FLAIR, MPRAGE, and DWI (*b*‐value = 0) acquisitions in the test stroke data. Similar to SWI and DWI *b*‐value $1200s/{\mathrm{mm}}^{2}$, appropriate selection of fine‐tuning hyperparameters improves reconstruction performance on R≈4 under‐sampled data. Figure S2 presents an example MPRAGE slice from the quantitative analysis of Figure S1.

**FIGURE 8:**
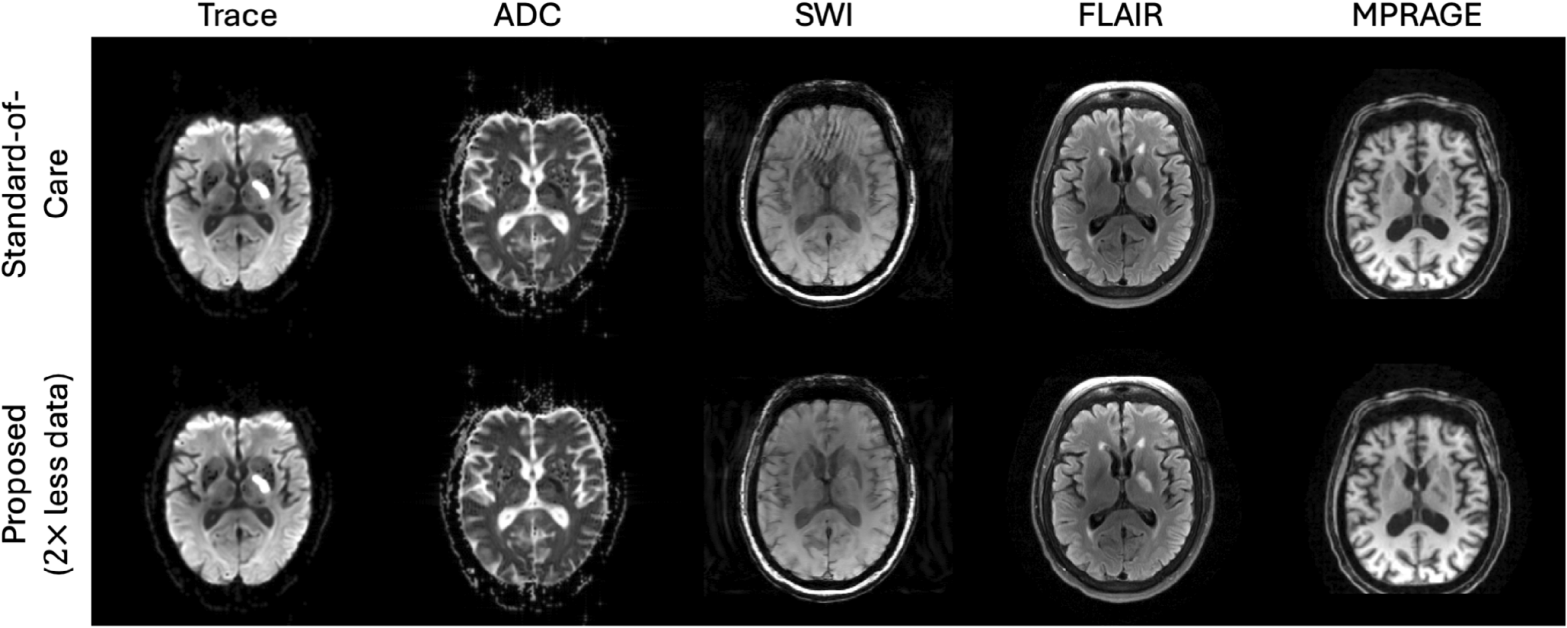
Representative images from a clinical stroke protocol are shown for a single subject, comparing standard‐of‐care reconstructions (top row) with images retrospectively under‐sampled by an additional factor of two and reconstructed using the proposed DPM‐based approach (bottom row). The protocol includes diffusion‐weighted imaging (shown as derived trace and apparent diffusion coefficient [ADC] maps), susceptibility‐weighted imaging [SWI], FLAIR, and MPRAGE. Across all contrasts, the proposed accelerated reconstructions preserve structural detail and contrast comparable to the standard‐of‐care. This subject was rated by Reader 1 (H.S.) in the blinded reader study, with the proposed reconstruction receiving the maximum score of 5 across all evaluated metrics and the standard‐of‐care reference receiving 5 on every metric except artifact level, where it received 4. This subject was a patient with ischemic stroke.

Figure 8 presents example stroke protocol images from the standard‐of‐care and images from the same protocol retrospectively under‐sampled by an additional factor of 2 and reconstructed with DPMs fine‐tuned with the proposed strategy. For DWI, we display derived trace and apparent diffusion coefficient (ADC) maps, commonly used in clinical stroke assessment, instead of displaying the raw DWI images directly. The displayed subject is a patient with ischemic stroke.

### 3.3 Clinical Reader Study

**FIGURE 9:**
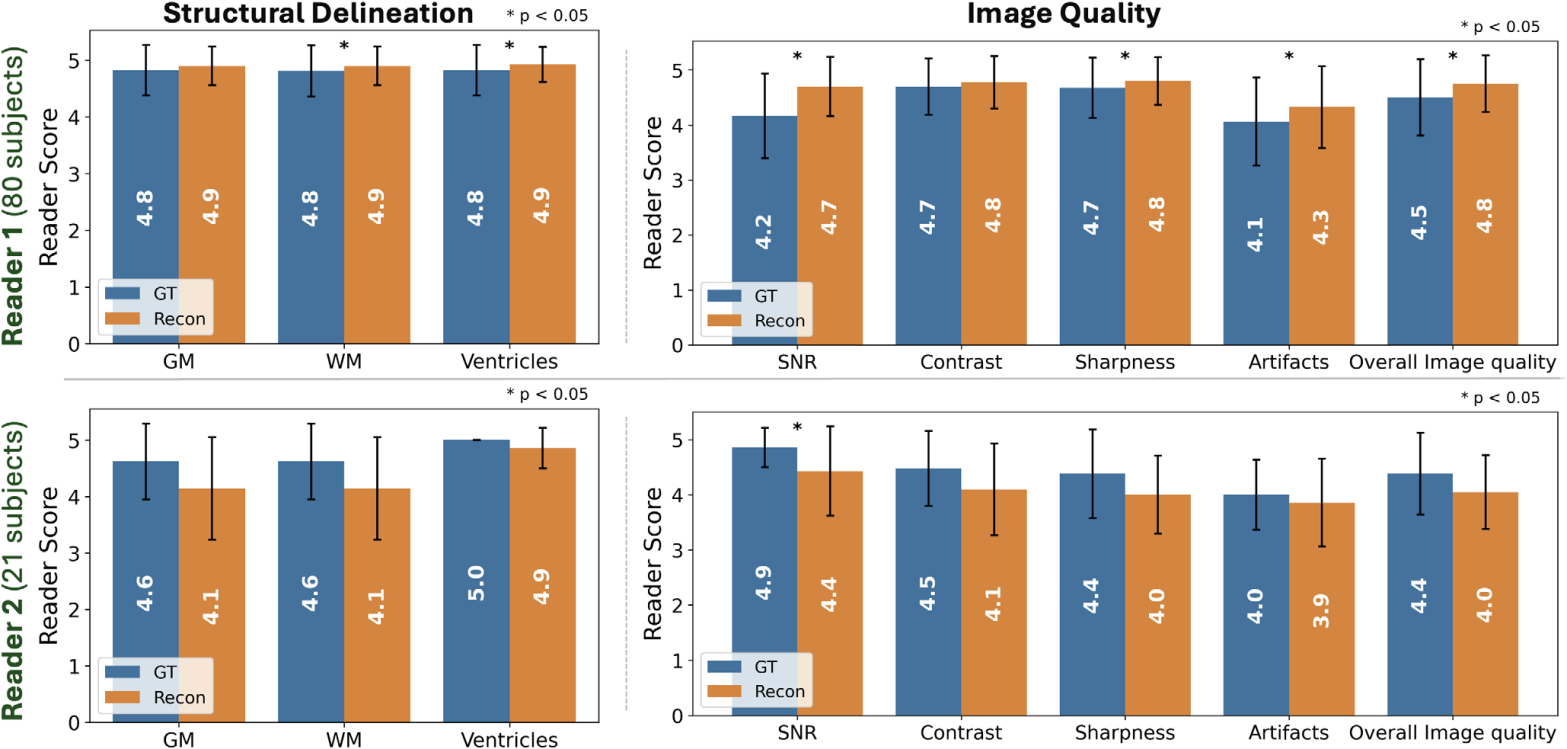
Mean reader scores for structural delineation (left) and image quality metrics (right) are shown for two board‐certified neuroradiologists evaluating full stroke‐protocol image sets reconstructed from standard‐of‐care data (GT) and from retrospectively accelerated data reconstructed using the proposed DPM‐based approach (Recon). The top row reports results from Reader 1, who evaluated all 80 subjects, while the bottom row reports results from Reader 2, who evaluated 21 subjects. For Reader 1, the proposed accelerated reconstructions achieved statistically significant higher scores for several image quality metrics and for white matter and ventricular delineation. For Reader 2, no significant differences were observed except for SNR, where the standard‐of‐care reconstruction scored higher; however, mean scores for both methods remained high across all categories.

Figure 9 summarizes the results of the blinded clinical reader study, in which two board‐certified neuroradiologists evaluated full stroke‐protocol image sets reconstructed from standard‐of‐care data and from retrospectively accelerated data, by a factor of 2, reconstructed using DPMs trained with the proposed approach.

The first reader evaluated image sets from all 80 subjects. For structural delineation, the mean scores [standard, accelerated] for gray matter, white matter, and ventricles were [4.8, 4.9], [4.8, 4.9], and [4.8, 4.9], respectively. No statistically significant difference was observed for gray matter delineation, while the proposed accelerated reconstruction achieved statistically significant improvements for white matter and ventricular delineation. For image quality metrics, mean scores [standard, accelerated] for SNR, contrast, sharpness, artifacts, and overall image quality were [4.2, 4.7], [4.7, 4.8], [4.7, 4.8], [4.1, 4.3], and [4.5, 4.8], respectively. The accelerated reconstruction demonstrated statistically significant improvements in SNR, sharpness, artifact level, and overall image quality, while no statistically significant difference was observed for contrast.

The second reader evaluated image sets from 21 subjects. For structural delineation, mean scores [standard, accelerated] for gray matter, white matter, and ventricles were [4.6, 4.1], [4.6, 4.1], and [5.0, 4.9], respectively, with no statistically significant differences observed. For image quality metrics, mean scores [standard, accelerated] for SNR, contrast, sharpness, artifacts, and overall image quality were [4.9, 4.4], [4.5, 4.1], [4.4, 4.0], [4.0, 3.9], and [4.4, 4.0], respectively. In this cohort, the standard‐of‐care reconstruction achieved a statistically significantly higher SNR score, while no statistically significant differences were observed for the remaining image quality metrics.

Inter‐rater reliability, as measured by Cohen's kappa coefficient, was fair agreement (0.21−0.40) for contrast, sharpness, artifacts, and overall image quality, slight agreement (0.00−0.20) for SNR, white matter, and gray matter, and poor agreement (<0.00) for ventricles.

Figure S3 displays two additional reader‐rated examples from the blinded reader study: (A) a subject for which both readers assigned low scores to several image quality metrics for both standard‐of‐care and proposed reconstructions, and (B) a subject for which the two readers diverged, with Reader 1 assigning the maximum score across all metrics and Reader 2 assigning lower scores. These examples directly visualize the inter‐rater variability quantified by the Cohen's kappa analysis reported below.

### 3.4 Experiments on Prospectively Under‐Sampled Data

**FIGURE 10:**
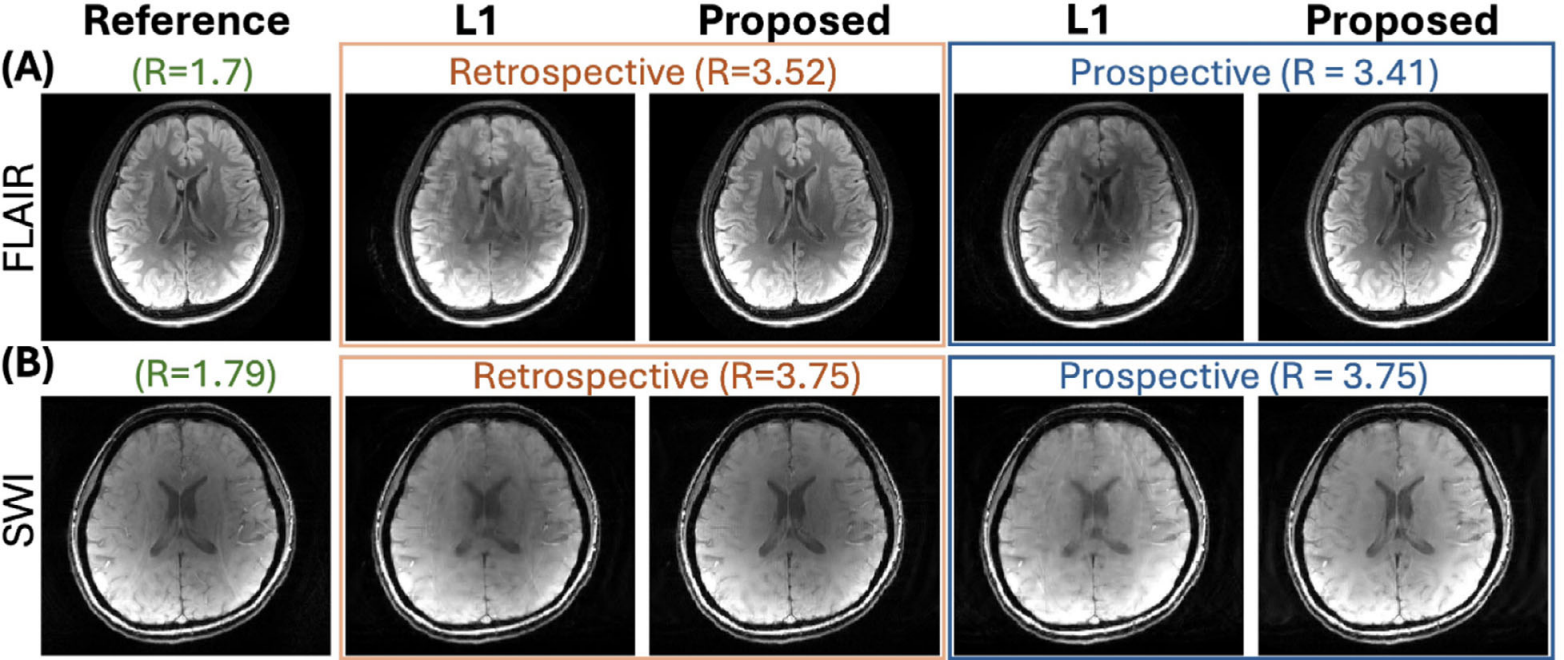
The proposed DPM, pre‐trained on fastMRI data and fine‐tuned on FLAIR or SWI data from 20 subjects, is applied to reconstruct both retrospectively and prospectively under‐sampled data from a healthy volunteer. For each contrast, the fully sampled reference, retrospective reconstructions, and prospective reconstructions are displayed along with their effective acceleration factors. Across both contrasts, the proposed method produces image quality approaching the standard‐of‐care reference, with similar quality between retrospective and prospective acquisitions, and the $\ell_{1}$‐wavelet shown as a reference for problem difficulty. This experiment is intended only to demonstrate the feasibility of prospective under‐sampling with the proposed reconstruction pipeline and should not be interpreted as benchmarking evidence against learned reconstruction baselines.

Figure 10 displays the model pre‐trained with $T_{1}$, $T_{2}$, and postcontrast $T_{1}$ fastMRI and fine‐tuned on FLAIR or SWI data from 20 subjects applied to reconstruct retrospectively and prospectively under‐sampled FLAIR and SWI data from a healthy subect. Comparison to an L1‐wavelet baseline shows that the model qualitatively reduces reconstruction artifacts. In addition, the reconstructions from the retrospectively and prospectively under‐sampled acquisitions show similar image quality.

## 4 Discussion

We proposed a simple and effective training strategy for DPMs that enables high‐quality MRI reconstruction in the data‐constrained settings studied here. The proposed approach combines large‐scale pre‐training on diverse external MRI datasets with targeted fine‐tuning on small, fully‐sampled datasets from the application of interest, with an astute selection of hyperparameters. In our controlled fastMRI experiments, DPMs trained with our strategy achieved reconstruction quality comparable to models trained on substantially larger target specific datasets. We further validated the approach on our clinically motivated stroke MRI application, where the proposed method generalized across multiple contrasts within the standard stroke imaging protocol. In our blinded clinical reader study of 80 subjects at a single clinical site, images retrospectively under‐sampled by an additional factor of two and reconstructed with DPMs fine‐tuned on 20 fully‐sampled subjects were rated as comparable to standard‐of‐care reconstructions for both structural delineation and image quality metrics.

We note that the individual building blocks of our approach, including score‐based diffusion models, approximate posterior sampling, and pre‐training with fine‐tuning, have each been developed and applied in prior work. Rather, the contribution of this work is empirical and application‐oriented: we systematically study how to combine these components effectively for accelerated stroke MRI reconstruction when target‐domain data are scarce, characterize the sensitivity of reconstruction quality to fine‐tuning and inference hyperparameters, and demonstrate clinical viability through a blinded reader study on stroke MRI.

In the controlled fastMRI FLAIR experiments, we observed a clear trade‐off between insufficient adaptation and overfitting during fine‐tuning. As shown in Figures 2 and 3, omitting fine‐tuning resulted in reconstructions that were poorly adapted to the target contrast, leading to suboptimal image quality. In contrast, excessive fine‐tuning, either through an increased number of epochs or an overly aggressive learning rate, degraded performance, consistent with overfitting to the limited size of the target dataset. Similar trends were observed when fine‐tuning to different contrasts in the clinical stroke protocol, where the optimal fine‐tuning configuration varied across target datasets. Together, these results highlight that, in these experimental settings, effective transfer of diffusion models to data‐limited MRI reconstruction tasks requires careful selection of fine‐tuning hyperparameters to balance contrast adaptation against overfitting or catastrophic forgetting [52].

The fastMRI setting further enabled a direct comparison of the proposed training strategy against alternative approaches with access to substantially larger amounts of target‐domain FLAIR data (Figure 4). Methods 1–3 incorporated FLAIR data from all 344 subjects during training and therefore represent upper‐bound baselines that rely on extensive target‐specific supervision. Despite being fine‐tuned using FLAIR data from only 20 subjects, the proposed approach (Method 4) achieved reconstruction quality comparable to these upper‐bound baselines. In contrast, Methods 5 and 6, which either trained exclusively on the limited 20‐subject FLAIR dataset or jointly on the large external dataset and the 20 subject FLAIR dataset, exhibited degraded performance relative to the proposed method. The cohort sizes here reflect data availability: pre‐training used all relevant fastMRI subjects (˜4000), and the 20‐subject fine‐tuning cohort mirrors the fully‐sampled clinical stroke dataset available at the time. We did not parametrically sweep performance against fine‐tuning or pre‐training cohort size or pre‐training class balance; these ablations are an interesting direction for future work.

Since Algorithm 1 represents an approximate posterior sampling procedure, fastMRI experiments in Figure 5 also characterized the effect of the data consistency weighting ζ on reconstruction performance. Higher acceleration rates corresponded to a larger optimal ζ. We suspect that less total data in the data consistency term results in a lower overall value relative to the prior term, so a larger weight needs to be placed on the consistency term with less available k‐space data.

Training DPMs for our limited stroke dataset motivated the proposed approach. Figures 6, 7, 8 show that the proposed fine‐tuning strategy provides a performance benefit for DPMs, with data from just 20 patients across the various contrasts used in the stroke MRI protocol, including FLAIR, MPRAGE, SWI, and DWI.

In the blinded clinical reader study (Figure 9), images reconstructed using the proposed DPM‐based approach from 2× less data were rated comparably to the standard‐of‐care reconstructions on the structural delineation and image quality metrics defined in this work. For the first reader, who evaluated all 80 subjects, the proposed approach achieved statistically significant, slightly higher scores than the standard‐of‐care in several categories, including SNR, artifact level, and overall image quality. In contrast, the second reader, who evaluated a smaller subset of 21 subjects, tended to rate the standard‐of‐care images slightly higher across most metrics. However, no statistically significant differences were observed for this reader except for SNR, where the standard‐of‐care achieved higher scores.

The reader study also has methodological limitations that temper claims of clinical equivalence or noninferiority. Inter‐rater reliability, measured by Cohen's kappa, ranged from poor (for ventricles) to fair (for contrast, sharpness, artifacts, and overall image quality), indicating that the two readers did not consistently agree at the level of individual image sets. Moreover, only reader H.S. evaluated all 80 image sets, while reader S.W. evaluated 21 subjects due to time constraints, limiting the degree to which the second reader's scores can be treated as independent confirmation of the first. Despite these inter‐reader differences, mean scores for both reconstruction approaches remained consistently high, with most metrics averaging near or above 4, suggesting that both approaches produced images the readers generally rated as clinically acceptable. A more definitive assessment of clinical equivalence or noninferiority would require a larger, multi‐site cohort evaluated in full by multiple independent readers within a formally designed noninferiority trial.

Reconstruction performance with the proposed method depends on several fine‐tuning and posterior‐sampling hyperparameters. Because the approach presumes a small fully‐sampled target cohort, this same cohort supplies a validation subset for hyperparameter selection; 5 stroke subjects were used for this purpose (Section 2.7). Across all contrasts studied (Figures 2, 5, 6, 7, and Figure S1), lower learning rates and moderate fine‐tuning durations yield the best performance, and the data‐consistency weight increases with acceleration, although the numerically optimal values differ across sequences. Deploying the approach to new sites, scanner vendors, or contrasts will likely require a similar small‐scale validation sweep, and developing heuristics or automatic hyperparameter selection strategies to reduce this tuning burden is a promising direction for future work.

All clinical stroke data were acquired at a single site on a single 3 T Siemens Vida scanner, and the overall stroke cohort remains modest (30 subjects for fine‐tuning, validation, and testing, and 80 additional subjects for the blinded reader study). We have therefore not yet characterized how the proposed approach behaves across sites, scanner vendors, field strengths, or patient populations differing in demographics, stroke subtype, or comorbidities. Similarly, while the fastMRI experiments are tightly controlled, they rely on a curated consortium dataset that may not reflect the full variability of routine clinical acquisitions. The results reported here should accordingly be interpreted as encouraging but preliminary, and multi‐site, multi‐vendor evaluation on larger and more diverse cohorts will be required before broader claims of generalizability can be made.

Most experiments in this work relied on retrospective under‐sampling, while the prospective evaluation was limited to data acquired from a healthy volunteer. The prospective figure is a feasibility demonstration that the fine‐tuned DPM works as intended on prospectively under‐sampled data. Accordingly, this experiment supports only the feasibility of prospective under‐sampling with the proposed pipeline; it should not be interpreted as benchmarking evidence against learned reconstruction baselines, including self‐supervised, zero‐shot, or test‐time adaptation methods, nor as support for claims of clinical deployment or methodological superiority. The L1‐wavelet shown alongside the proposed reconstruction is a classical‐reconstruction reference that conveys the difficulty of the under‐sampled inverse problem, analogous to how zero‐filled reconstructions are commonly displayed, rather than a competing learned baseline. More rigorous benchmarking against strong supervised regimes appears in Figure 4. Future work will therefore focus on validating the proposed approach using prospectively under‐sampled clinical stroke MRI acquisitions to more fully assess performance. In addition, future reader studies will evaluate the impact of the proposed reconstructions on downstream clinical interpretation and decision‐making relative to standard‐of‐care images. Finally, the current posterior sampling implementation incurs substantially longer reconstruction times per slice compared to conventional parallel imaging and end‐to‐end learning‐based methods. Future work will investigate more efficient implementations and improved posterior sampling strategies to reduce reconstruction time and facilitate practical clinical deployment.

Several diffusion‐based approaches have been proposed to address target‐domain adaptation in MRI reconstruction, including per‐scan test‐time prior adaptation [30, 31, 32] and self‐supervised training on under‐sampled target‐domain k‐space [33]. Our approach addresses a complementary setting: it targets applications where a small fully‐sampled target cohort can be collected, yielding a single fine‐tuned model that is deployed across new subjects at inference without additional per‐subject adaptation, as in our 20‐subject stroke fine‐tuning cohort. These methods are therefore complementary to ours rather than direct alternatives; the fine‐tuned DPM could itself serve as the starting point for per‐scan inference‐time adaptation or for subsequent self‐supervised refinement on additional under‐sampled data. A natural direction for future work is to integrate these techniques with the proposed training strategy and characterize, through controlled ablations, how different combinations of adaptation mechanisms affect reconstruction quality. We emphasize that this study does not include per‐scan diffusion adaptation, self‐supervised diffusion reconstruction, or zero‐shot out‐of‐distribution methods as quantitative baselines; the reported results should therefore not be interpreted as evidence that the proposed fine‐tuning strategy is methodologically superior to these approaches. The quantitative comparisons in this work (Figure 4) are confined to alternative target‐domain data regimes, and our contribution remains the empirical finding that large‐scale pre‐training combined with small‐sample fine‐tuning is a practical strategy for accelerated stroke MRI under limited target‐domain data.

A related direction concerns parameter‐efficient fine‐tuning strategies that adapt a pre‐trained model without updating all of its weights, including low‐rank adaptation (LoRA) [53, 54], adapter modules [55], hypernetworks [56], and partial fine‐tuning of selected layers [57]. In this work, we adapt the entire network using a reduced learning rate and limited number of epochs, motivated by the computational flexibility of full fine‐tuning at this model scale and the empirical reconstruction performance we observed. Future work could explore whether these parameter‐efficient alternatives further improve reconstruction quality, reduce fine‐tuning data requirements, or facilitate scaling to additional target contrasts, sites, and scanner vendors.

A related question is whether the multiple contrasts of the stroke protocol should be fine‐tuned jointly or separately. Although the contrast‐conditioning architecture (Section 2.2) supports joint multi‐contrast fine‐tuning in principle, we fine‐tuned a separate model per sequence because the optimal fine‐tuning learning rate and number of epochs might differ across contrasts, letting each contrast use its empirically best hyperparameters. Joint multi‐contrast fine‐tuning, and the cross‐contrast knowledge transfer it might enable, is a natural direction for future work.

## Funding

This work was supported by NSF IFML (2019844), NSF (CCF‐2239687) (CAREER) and Joint Center for Computational Oncology (JCCO) Postdoctoral Fellowship.

## Supporting information


**Table S1:** Using our method for training DPMs, average NRMSE on fastMRI FLAIR validation for the best combination of data‐consistency step‐size (ζ) and number of posterior sampling steps (N) for different acceleration rates. As the acceleration rate increases, ζ also increases.
**Figure S1:** Experiments analyzing the influence of fine‐tuning epochs and learning rate when training a DPM with our proposed approach for reconstruction of additional FLAIR, MPRAGE, and DWI (*b*‐value = 0) acquisitions in the test stroke data. Like SWI and DWI, appropriate selection of fine‐tuning hyperparameters improves reconstruction performance on R≈4 under‐sampled data.
**Figure S2:** (A) NRMSE of MPRAGE reconstructions averaged over five validation subjects from data retrospectively accelerated by R≈3.75. (B) Fully‐sampled reference. (C) Zoomed‐in reconstructions obtained with fixed epochs and varying learning rate and (D) fixed learning rate and varying epochs. Like the fastMRI FLAIR experiments, insufficient or excessive fine‐tuning duration and higher learning rates consistently result in worse reconstructions, demonstrating that the proposed training strategy is effective for clinical stroke MPRAGE data.
**Figure S3:** Additional reader‐study examples in which both readers rated the image sets low on their rating or disagreed. Each panel shows representative set of slices from the stroke protocol reconstructed with the standard‐of‐care (GT) or proposed approach (Recon). Per‐image set ratings from two expert readers (R1, R2) are listed for SNR, Con (contrast), Sh (sharpness), Art (artifacts), Ov (overall image quality). (A) Concordant low‐quality case where both readers assigned predominantly 2–3 ratings. (B) Discordant case where R1 scored Recon 5 across all metrics while reader scored Recon 3.
**Figure S4:** Contrast‐conditioning pathway of the diffusion model. Each contrast's one‐hot vector (dimension 9) is mapped by a fully‐connected network to a 512‐dimensional contrast embedding. This embedding is added to the noise‐level (*σ*) embedding and processed by a shared two‐layer fully‐connected network with SiLU activations. The resulting conditioning vector is supplied to every residual block of the U‐Net, where a learned linear (affine) layer maps it to a per‐channel bias that is added to the block's feature maps prior to group normalization—modulating activations through the same pathway as the noise‐level embedding, rather than being concatenated as an extra channel to the convolutional layers. This follows the class‐conditioning mechanism of EDM.

## Data Availability

Research data are not shared.

## References

[1] R. Heckel , M. Jacob , A. Chaudhari , O. Perlman , and E. Shimron , “Deep Learning for Accelerated and Robust Mri Reconstruction,” Magnetic Resonance Materials in Physics, Biology and Medicine 37 (2024): 335–368. 10.1007/s10334-024-01173-8 PMC11316714 39042206

[2] K. Hammernik , T. Küstner , B. Yaman , et al., “Physics‐Driven Deep Learning for Computational Magnetic Resonance Imaging: Combining Physics and Machine Learning for Improved Medical Imaging,” IEEE Signal Processing Magazine 40, no. 1 (2023): 98–114. 37304755 10.1109/msp.2022.3215288 PMC10249732

[3] M. Muckley , B. Riemenschneider , A. Radmanesh , et al., “Results of the 2020 FastMRI Challenge for Machine Learning MR Image Reconstruction,” IEEE Transactions on Medical Imaging 40 (2021): 2306–2317. 33929957 10.1109/TMI.2021.3075856 PMC8428775

[4] J. Lyu , C. Qin , S. Wang , et al., “The State‐Of‐The‐Art in Cardiac Mri Reconstruction: Results of the CMRxRecon Challenge in MICCAI 2023,” Medical Image Analysis 101 (2025): 103485. 39946779 10.1016/j.media.2025.103485

[5] A. D. Desai , A. M. Schmidt , E. B. Rubin , et al., “Skm‐Tea: A Dataset for Accelerated MRI Reconstruction With Dense Image Labels for Quantitative Clinical Evaluation,” *Advances in Neural Information Processing Systems (Datasets and Benchmarks Track)* (2021).

[6] R. Bommasani , D. A. Hudson , E. Adeli , et al., “On the Opportunities and Risks of Foundation Models,” *arXiv* (2021).

[7] C. Zhou , Q. Li , C. Li , et al., “A Comprehensive Survey on Pretrained Foundation Models: A History From Bert to Chatgpt,” International Journal of Machine Learning and Cybernetics 16 (2024): 9851–9915.

[8] W. X. Zhao , K. Zhou , J. Li , et al., “A Survey of Large Language Models,” *arXiv* (2023).

[9] A. Radford , J. W. Kim , C. Hallacy , et al., “Learning Transferable Visual Models From Natural Language Supervision,” *Proceedings of Machine Learning Research* (2021).

[10] A. Kirillov , E. Mintun , N. Ravi , et al., “Segment Anything,” *IEEE/CVF International Conference on Computer Vision (ICCV)* (2023).

[11] C. Ma , W. Tan , R. He , and B. Yan , “Pretraining a Foundation Model for Generalizable Fluorescence Microscopy‐Based Image Restoration,” Nature Methods 21 (2024): 1558–1567. 38609490 10.1038/s41592-024-02244-3

[12] Z. Huang , F. Bianchi , M. Yuksekgonul , T. J. Montine , and J. Zou , “A Visual–Language Foundation Model for Pathology Image Analysis Using Medical Twitter,” Nature Medicine 29 (2023): 2307–2316. 10.1038/s41591-023-02504-3 37592105

[13] Y. Zhou , M. A. Chia , S. K. Wagner , et al., “A Foundation Model for Generalizable Disease Detection From Retinal Images,” Nature 622 (2023): 156–163. 37704728 10.1038/s41586-023-06555-x PMC10550819

[14] M. Moor , O. Banerjee , Z. S. H. Abad , et al., “Foundation Models for Generalist Medical Artificial Intelligence,” Nature 616 (2023): 259–265. 37045921 10.1038/s41586-023-05881-4

[15] D. Birenbaum , L. Bancroft , and G. Felsberg , “Imaging in Acute Stroke,” Western Journal of Emergency Medicine 12 (2011): 67–76. 21694755 PMC3088377

[16] C. M. Rapillo , V. Dunet , S. Pistocchi , et al., “Moving From Ct to Mri Paradigm in Acute Ischemic Stroke: Feasibility, Effects on Stroke Diagnosis and Long‐Term Outcomes,” Stroke 55 (2024): 1329–1338. 38488367 10.1161/STROKEAHA.123.045154 PMC11045552

[17] S. Altmann , N. F. Grauhan , L. Brockstedt , et al., “Ultrafast Brain Mri With Deep Learning Reconstruction for Suspected Acute Ischemic Stroke,” Radiology 310, no. 2 (2024): e231938. 38376403 10.1148/radiol.231938

[18] M. Akçakaya , S. Moeller , S. Weingärtner , and K. Uğurbil , “Scan‐Specific Robust Artificial‐Neural‐Networks for k‐Space Interpolation (Raki) Reconstruction: Database‐Free Deep Learning for Fast Imaging,” Magnetic Resonance in Medicine 81 (2019): 439–453. 30277269 10.1002/mrm.27420 PMC6258345

[19] Y. Arefeen , O. Beker , J. Cho , E. Adalsteinsson , and B. Bilgic , “Scan‐Specific Artifact Reduction in k‐Space (Spark) Neural Networks Synergize With Physics‐Based Reconstruction to Accelerate Mri,” Magnetic Resonance in Medicine 87 (2022): 764–780. 34601751 10.1002/mrm.29036 PMC8627503

[20] R. Feng , Q. Wu , J. Feng , et al., “Imjense: Scan‐Specific Implicit Representation for Joint Coil Sensitivity and Image Estimation in Parallel Mri,” IEEE Transactions on Medical Imaging 43 (2024): 1539–1553. 38090839 10.1109/TMI.2023.3342156

[21] M. Darestani and R. Heckel , “Accelerated Mri With Un‐Trained Neural Networks,” IEEE Transactions on Computational Imaging 7 (2021): 724–733.

[22] B. Yaman , S. A. H. Hosseini , and M. Akçakaya , “Zero‐Shot Self‐Supervised Learning for Mri Reconstruction,” *International Conference on Learning Representations* (2022).

[23] B. Yaman , S. Amir , H. Hosseini , et al., “Self‐Supervised Learning of Physics‐Guided Reconstruction Neural Networks Without Fully Sampled Reference Data,” Magnetic Resonance in Medicine 84 (2020): 3172–3191. 32614100 10.1002/mrm.28378 PMC7811359

[24] C. Millard and M. Chiew , “A Theoretical Framework for Self‐Supervised MR Image Reconstruction Using Sub‐Sampling via Variable Density Noisier2Noise,” IEEE Transactions on Computational Imaging 9 (2023): 707–720. 37600280 10.1109/TCI.2023.3299212 PMC7614963

[25] S. Xu , M. Früh , K. Hammernik , et al., “Self‐Supervised Feature Learning for Cardiac Cine Mr Image Reconstruction,” IEEE Transactions on Medical Imaging 44, no. 9 (2025): 3858–3869. 40408221 10.1109/TMI.2025.3570226

[26] V. Spieker , H. Eichhorn , W. Huang , et al., “Pisco: Self‐Supervised k‐Space Regularization for Improved Neural Implicit k‐Space Representations of Dynamic Mri,” Medical Image Analysis 109 (2026): 103890. 41371062 10.1016/j.media.2025.103890

[27] A. D. Desai , B. M. Ozturkler , C. M. Sandino , et al., “Noise2recon: Enabling Snr‐Robust Mri Reconstruction With Semi‐Supervised and Self‐Supervised Learning,” Magnetic Resonance in Medicine 90 (2023): 2052–2070. 37427449 10.1002/mrm.29759

[28] A. Wang , S. McDonagh , and M. Davies , “Benchmarking Self‐Supervised Learning Methods for Accelerated MRI Reconstruction,” *arXiv* (2025).

[29] M. Z. Darestani , J. Liu , and R. Heckel , Test‐Time Training Can Close the Natural Distribution Shift Performance Gap in Deep Learning Based Compressed Sensing (ICML, 2022).

[30] A. Güngör , S. U. H. Dar , Ş. Öztürk , et al., “Adaptive Diffusion Priors for Accelerated MRI Reconstruction,” Medical Image Analysis 88 (2023): 102872. 37384951 10.1016/j.media.2023.102872

[31] R. Barbano , A. Denker , H. Chung , et al., “Steerable Conditional Diffusion for Out‐Of‐Distribution Adaptation in Medical Image Reconstruction,” IEEE Transactions on Medical Imaging 44 (2025): 2093–2104. 40030859 10.1109/TMI.2024.3524797

[32] H. Chung and J. C. Ye , “Deep Diffusion Image Prior for Efficient OOD Adaptation in 3D Inverse Problems,” *in European Conference on Computer Vision (ECCV)* (2024).

[33] Y. Korkmaz , T. Çukur , and V. M. Patel , “Self‐Supervised MRI Reconstruction With Unrolled Diffusion Models,” *in Medical Image Computing and Computer Assisted Intervention (MICCAI)* (2023).

[34] J. Parmar , S. Satheesh , M. Patwary , M. Shoeybi , and B. Catanzaro , “Reuse, Don't Retrain: A Recipe for Continued Pretraining of Language Models,” arXiv (2024).

[35] K. Lin and R. Heckel , “Robustness of Deep Learning for Accelerated Mri: Benefits of Diverse Training Data,” *International Conference on Machine Learning* (2024).

[36] Y. Song , J. Sohl‐Dickstein , D. P. Kingma , A. Kumar , S. Ermon , and B. Poole , “Score‐Based Generative Modeling Through Stochastic Differential Equations,” *International Conference on Learning Representations* (2021).

[37] H. Chung and J. C. Ye , “Score‐Based Diffusion Models for Accelerated Mri,” Medical Image Analysis 80 (2022): 102479. 35696876 10.1016/j.media.2022.102479

[38] H. Chung , J. Kim , M. T. Mccann , M. L. Klasky , and J. C. Ye , “Diffusion Posterior Sampling for General Noisy Inverse Problems,” *International Conference on Learning Representations* (2023).

[39] A. Jalal , M. Arvinte , G. Daras , E. Price , A. G. Dimakis , and J. I. Tamir , “Robust Compressed Sensing Mri With Deep Generative Priors,” *Advances in Neural Information Processing Systems* (2021).

[40] G. Luo , M. Blumenthal , M. Heide , and M. Uecker , “Bayesian Mri Reconstruction With Joint Uncertainty Estimation Using Diffusion Models,” Magnetic Resonance in Medicine 90 (2023): 295–311. 36912453 10.1002/mrm.29624

[41] L. Yang , Z. Zhang , Y. Song , et al., “Diffusion Models: A Comprehensive Survey of Methods and Applications,” ACM Computing Surveys 56 (2023): 1–39.

[42] J. Ho , A. Jain , and P. Abbeel , “Denoising Diffusion Probabilistic Models,” *Advances in Neural Information Processing Systems* (2020).

[43] H. Zheng , W. Chu , B. Zhang , et al., “Inversebench: Benchmarking Plug‐And‐Play Diffusion Priors for Inverse Problems in Physical Sciences,” *International Conference on Learning Representations* (2025).

[44] G. Luo , N. Zhao , W. Jiang , E. S. Hui , and P. Cao , “Mri Reconstruction Using Deep Bayesian Estimation,” Magnetic Resonance in Medicine 84 (2020): 2246–2261. 32274850 10.1002/mrm.28274

[45] K. Tezcan , C. Baumgartner , R. Luechinger , K. Pruessmann , and E. Konukoglu , “Mr Image Reconstruction Using Deep Density Priors,” IEEE Transactions on Medical Imaging 38 (2019): 1633–1642. 30571618 10.1109/TMI.2018.2887072

[46] T. Karras , M. Aittala , T. Aila , and S. Laine , “Elucidating the Design Space of Diffusion‐Based Generative Models,” Advances in Neural Information Processing Systems 35 (2022): 26565–26577.

[47] Y. Arefeen , B. Levac , B. Patel , C. Ho , and J. I. Tamir , “Diffusion Probabilistic Generative Models for Accelerated, In‐Nicu Permanent Magnet Neonatal Mri,” Magnetic Resonance in Medicine 94 (2025): 1546–1562. 40528527 10.1002/mrm.30585 PMC12309888

[48] M. Terris , S. Hurault , M. Song , and J. Tachella , Reconstruct Anything Model a Lightweight Foundation Model for Computational Imaging (ICLR, 2026).

[49] R. Feng , X. He , R. Mercer , Z. Stewart , and F. Liu , “On the Utility of Foundation Models for Fast Mri: Vision‐Language‐Guided Image Reconstruction,” Magnetic Resonance in Medicine 96, no. 2 (2026): 943–959. 41947281 10.1002/mrm.70374 PMC13499591

[50] H. Li , M. Chiew , I. Dragonu , P. Jezzard , and T. W. Okell , “Few‐Shot Learning for Highly Accelerated 3d Time‐Of‐Flight Mra Reconstruction,” Magnetic Resonance in Medicine 95, no. 2 (2026): 770–786. 40931540 10.1002/mrm.70072 PMC12681298

[51] M. Uecker , F. Ong , J. I. Tamir , et al., “Berkeley Advanced Reconstruction Toolbox,” *Proceedings of the International Society of Magnetic Resonance in Medicine* (2015).

[52] J. Kirkpatrick , R. Pascanu , N. Rabinowitz , et al., “Overcoming Catastrophic Forgetting in Neural Networks,” Proceedings of the National Academy of Sciences of the United States of America 114, no. 13 (2017): 3521–3526. 28292907 10.1073/pnas.1611835114 PMC5380101

[53] E. J. Hu , Y. Shen , P. Wallis , et al., “LoRA: Low‐Rank Adaptation of Large Language Models,” *in International Conference on Learning Representations (ICLR)* (2022).

[54] R. Gandikota , J. Materzyńska , T. Zhou , A. Torralba , and D. Bau , “Concept Sliders: LoRA Adaptors for Precise Control in Diffusion Models,” *in European Conference on Computer Vision (ECCV)* (2024).

[55] N. Houlsby , A. Giurgiu , S. Jastrzebski , et al., “Parameter‐Efficient Transfer Learning for NLP,” *in International Conference on Machine Learning (ICML)* (2019).

[56] D. Ha , A. M. Dai , and Q. V. Le , “HyperNetworks,” *in International Conference on Learning Representations (ICLR)* (2017).

[57] J. Yosinski , J. Clune , Y. Bengio , and H. Lipson , “How Transferable Are Features in Deep Neural Networks?” *in Advances in Neural Information Processing Systems (NeurIPS)* (2014).

