# Supplementary material for: Accelerating Stroke MRI With Diffusion Probabilistic Models Through Large‐Scale Pre‐Training and Target‐Specific Fine‐Tuning
Source: Magn Reson Med. 2026 Jul 9;96(5):2396–411. doi: 10.1002/mrm.70501 (PMC13527248; doi:10.1002/mrm.70501)
Supplement: Supplementary file 1 [file MRM-96-2396-s001.docx]

**Supporting Information**

**Supporting Table S1:** Using our method for training DPMs, average NRMSE on fastMRI FLAIR validation for the best combination of data-consistency step-size ($\zeta)$ and number of posterior sampling steps $(N)$ for different acceleration rates. As the acceleration rate increases, $\zeta$also increases.

**Supporting Figure S1:** Experiments analyzing the influence of fine-tuning epochs and learning rate when training a DPM with our proposed approach for reconstruction of additional FLAIR, MPRAGE, and DWI (b-value = 0) acquisitions in the test stroke data. Like SWI and DWI, appropriate selection of fine-tuning hyperparameters improves reconstruction performance on $R\approx4$under-sampled data.

**Supporting Figure S2:** (A) NRMSE of MPRAGE reconstructions averaged over five validation subjects from data retrospectively accelerated by $R\approx3.75$. (B) Fully-sampled reference. (C) Zoomed-in reconstructions obtained with fixed epochs and varying learning rate and (D) fixed learning rate and varying epochs. Like the fastMRI FLAIR experiments, insufficient or excessive fine-tuning duration and higher learning rates consistently result in worse reconstructions, demonstrating that the proposed training strategy is effective for clinical stroke MPRAGE data.

**Supporting Figure S3:** Additional reader-study examples in which both readers rated the image sets low on their rating or disagreed. Each panel shows representative set of slices from the stroke protocol reconstructed with the standard-of-care (GT) or proposed approach (Recon). Per-image set ratings from two expert readers (R1,R2) are listed for SNR, Con (contrast), Sh (sharpness), Art (artifacts), Ov (overall image quality). (A) Concordant low-quality case where both readers assigned predominantly 2-3 ratings. (B) Discordant case where R1 scored Recon 5 across all metrics while reader scored Recon 3.

**Supporting Figure S4:** Contrast-conditioning pathway of the diffusion model. Each contrast's one-hot vector (dimension 9) is mapped by a fully-connected network to a 512-dimensional contrast embedding. This embedding is added to the noise-level (σ) embedding and processed by a shared two-layer fully-connected network with SiLU activations. The resulting conditioning vector is supplied to every residual block of the U-Net, where a learned linear (affine) layer maps it to a per-channel bias that is added to the block's feature maps prior to group normalization — modulating activations through the same pathway as the noise-level embedding, rather than being concatenated as an extra channel to the convolutional layers. This follows the class-conditioning mechanism of EDM.
